# Woodpeckers can act as dispersal vectors for fungi, plants, and microorganisms

**DOI:** 10.1002/ece3.7648

**Published:** 2021-05-13

**Authors:** Niko R. Johansson, Ulla Kaasalainen, Jouko Rikkinen

**Affiliations:** ^1^ Finnish Museum of Natural History University of Helsinki Helsinki Finland; ^2^ Organismal and Evolutionary Biology Research Programme Faculty of Biological and Environmental Sciences University of Helsinki Helsinki Finland; ^3^ Department of Geobiology University of Göttingen Göttingen Germany

**Keywords:** collection‐based research, dispersal, epizoochory, lichen, spore, woodpecker

## Abstract

Bird‐mediated dispersal is presumed to be important in the dissemination of many different types of organisms, but concrete evidence remains scarce. This is especially true for biota producing microscopic propagules. Tree‐dwelling birds, such as woodpeckers, would seem to represent ideal dispersal vectors for organisms growing on standing tree trunks such as epiphytic lichens and fungi. Here, we utilize bird natural history collections as a novel source of data for studying dispersal ecology of plants, fungi, and microorganisms. We screened freshly preserved specimens of three Finnish woodpecker species for microscopic propagules. Samples were taken from bird feet, and chest and tail feathers. Propagules were extracted using a sonication–centrifugation protocol, and the material obtained was studied using light microscopy. Diverse biological material was recovered from all specimens of all bird species, from all positions sampled. Most abundant categories of discovered biological material included bryophyte fragments, fungal spores, and vegetative propagules of lichens. Also, freshwater diatoms, bryophyte spores, algal cells, testate amebae, rotifers, nematodes, pollen, and insect scales were identified. The method developed here is applicable to living specimens as well, making it a versatile tool for further research. Our findings highlight the potential of bird‐mediated dispersal for diverse organisms and showcase the use of natural history collections in ecological research.

## Graphical abstract

Woodpeckers are ideal candidates for bird‐mediated dispersal for a diverse set of organisms, especially epiphytes. We report high loads of diverse biological propagules extracted from feathers and feet of woodpecker specimens from natural history collections. The study highlights the potential of birds as dispersal vectors of lichens, fungi, bryophytes, and more.

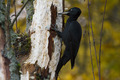

## 1 INTRODUCTION

Dispersal is a fundamental factor that shapes the distribution of organisms in nature. It is also difficult to observe and study, especially with microorganisms and larger organisms that produce microscopic propagules (Tesson et al., 2016). Often, microscopic taxa are thought not to have dispersal limitations, with their propagules being ubiquitously dispersed via air or water currents globally (Finlay, 2002). Recent findings in molecular microbial biogeography have provided contrary evidence to this “everything is everywhere” hypothesis (Aguilar et al., 2014; Pinseel et al., 2020; Ribeiro et al., 2018). While long‐distance dispersal undoubtedly is the best explanation for many observed species distribution patterns (Garrido‐Benavent & Perez‐Ortega, 2017; Lewis et al., 2014), mechanisms that could facilitate such dispersal events are not thoroughly investigated.

Various animals facilitate the dispersal of other organisms ranging from microbes and invertebrates to plants and fungi. Birds particularly as highly mobile and often migratory animals have been hypothesized to facilitate long‐distance dispersal, as noted already by Darwin (1859). However, actual empirical evidence is still very limited for many taxa, for both vectors and dispersers (Garrido‐Benavent & Perez‐Ortega, 2017; Viana et al., 2016). Previous work has often focused on endozoochory, for example, dispersal internally via ingested seeds or other dispersal units (Costa et al., 2014). However, for many taxa not being a part of bird diet, dispersal via external attachment (epi‐ or ectozoochory) seems more logical. For example, among lichenized fungi, observational studies of bird‐mediated dispersal are mainly restricted to thallus fragments attached to bird feet (Bailey & James, 1979) and the use of lichen thalli as nest‐building material (Graves & Forno, 2018; Parnikoza et al., 2018). Observational evidence of microscopic lichen propagules, such as spores or vegetative dispersal units, being attached to bird plumage is meager (Lewis, Behling, et al., 2014; Warner & French, 1970), and a recent review of bird–fungal interactions did not mention lichens at all (Elliott et al., 2019).

Bird‐mediated dispersal could potentially affect a very diverse set of taxa, but the relatively few previous studies have usually focused on a single group, such as certain bryophytes (Chmielewski & Eppley, 2019). Lewis, Behling, et al. (2014) identified bryophyte diaspores and fungal spores from plumage of migrant shorebird chest feathers. Chmielewski and Eppley (2019) surveyed different groups of forest birds and identified viable bryophyte material from bird feet and tail feathers. Thrushes, corvids and woodpeckers were identified as groups with high loads of bryophyte propagules, indicating that these birds might also transport propagules of other organisms. Woodpeckers as cavity excavators are in close contact to open wood and bark, which represent a primary substrate for many organisms. Woodpeckers also have rigid, balancing tails that scrape trunk surfaces as they forage (Jackson, 1971). Indeed, they have previously been shown to play a role in dispersing some plant pathogenic fungi (Heald & Studhalter, 1913), wood‐inhabiting basidiomycetes (Elliott et al., 2019; Jusino, 2014; Jusino et al., 2016), and bryophytes (Chmielewski & Eppley, 2019), thus making them prime suspects as dispersal vectors for other taxa, including epiphytic lichens and fungi.

Results of searches for microscopic propagules in bird feathers aiming to identify the full range of taxa encountered have apparently not been previously published. However, the potential implications of birds as dispersal vectors in various groups (including vascular plant seeds, fern and bryophyte spores and fragments, and propagules of lichenized and nonlichenized fungi, algae, and microorganisms) are vast (Viana et al., 2016). These range from explaining biogeographical patterns (Biersma et al., 2017; Garrido‐Benavent & Perez‐Ortega, 2017; Lovas‐Kiss et al., 2019) to distribution dynamics of invasive species (Lovas‐Kiss et al., 2019; Molefe et al., 2020; Russo et al., 2019) and the spread of pathogens (Alfonzo et al., 2013; Hubálek, 2004), both in terrestrial and in aquatic ecosystems (Coughlan et al., 2017; Hessen et al., 2019). In fragmented landscapes, understanding the role of dispersal and connectivity in conservation is important (Keeley et al., 2018; Trakhtenbrot et al., 2005), but due to a general lack of information, modeling approaches and connectivity measures rarely take into account the potential of animal‐mediated dispersal (Rogers et al., 2019; Viana et al., 2016).

Natural history collections are a key source of biological data. Bird collections have traditionally been used not only in taxonomic studies and as teaching material, but recently also in many other innovative contexts (Webster, 2017), such as thermal insulation (Graveley et al., 2020), toxicology (Bond & Lavers, 2020), and stable isotope analysis (English et al., 2018). Here, we utilize bird collections as a resource for studying dispersal ecology. Museum specimens can be utilized in a parallel way to screening living birds, without the associated difficulties regarding field logistics and ethical issues. In this study, we describe a novel method and perspective for using bird specimens in natural history collections for the study of dispersal in a wide range of taxa. More specifically, we explore the diversity of biological material attached to woodpeckers and identify encountered propagules, with special focus on epiphytic organisms.

## 2 MATERIALS AND METHODS

**FIGURE 1:**
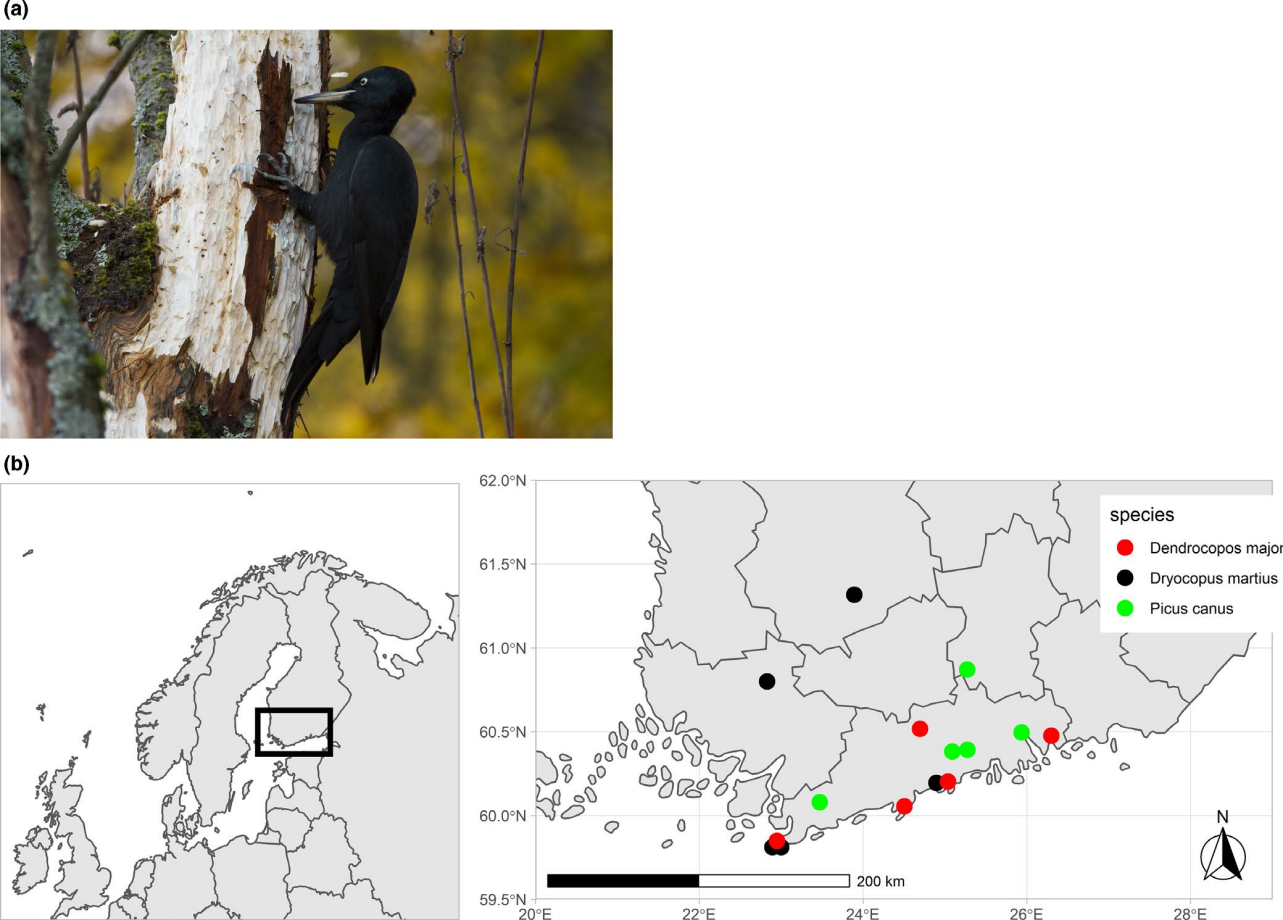
(a) *Dryocopus martius*, one of our study species. Note the intimate contact with the wood substrate. Picture by Jon Rikberg. (b) A map of localities where woodpecker samples were collected (*N* = 15)

Bird specimens were obtained from recent accessions to the collections of the Finnish Museum of Natural History (LUOMUS). We sampled altogether 15 specimens of three woodpecker species native to Finland (*Dryocopus martius* (Figure 1a), Dendrocopos major, and Picus canus, five specimens of each species). The localities of the specimens used can be seen in Figure 1b. Bird specimens were selected based on collecting date, cause of death, body condition, and postaccession preparation. Only recently acquired specimens that had been removed from their natural setting fast, for example, due to a window collision or bird ringing accident, were chosen to avoid a risk of postmortem contamination. All specimens were in good physical condition and had been quickly frozen after collection, wrapped in clean newspaper, shipped to the museum and then incorporated into the collections, labeled, bagged in airtight plastic bags, and frozen for further study. The birds had not been washed or otherwise treated before sampling. More information on the individual bird specimens is given in Appendix S1.

All bird specimens selected for analysis were processed as follows. First, the specimens were removed from their bags and thawed. This was done to avoid condensation of moisture on the thawing birds. Three different cotton swabs were used to sample the chest feathers, tail feathers, and feet of each bird, respectively. For feather sampling, the swab was run proximally multiple times across multiple feathers, and in the tail feathers, especially any visible dusty material attached to the underside of the pin of the feather was targeted. Feet were sampled in a circular motion on the underside of the digits, including under the claws. In addition, a single top‐layer chest feather and a 2‐cm fragment from the tip of one tail feather (rectrix 1 or 2) were removed for detailed analysis.

Collected cotton swabs and feathers were suspended in 500 μl of Milli‐Q water in sterile 1.5‐ml Eppendorf tubes. Tubes were vortexed briefly and placed in a sonication bath for 3 min to dislodge biological material from the cotton/feathers. After sonication, tubes were again briefly vortexed followed by 5‐min centrifugation (16,000 *g*). Feathers/swab tips were then carefully removed from the tubes with forceps, avoiding disruption of the potential pellet. This was followed by 3‐min centrifugation (16,000 *g*), after which most of the supernatant was removed. The remaining suspension (10–20 μl) was briefly vortexed, spun down, and inspected and photographed under phase‐contrast microscopy (Leica Microsystems, Wetzlar, Germany). Biological material was identified as accurately as possible, and the abundances of main categories of propagules encountered were roughly estimated. As direct counts were impossible due to the large amount of material, the amount of the main three categories was semiquantitatively estimated, ranking from no material to few (1–5) units to multiple (5+) units. The combined abundance estimates of the three main disperser categories were compared using one‐sided Wilcoxon rank sum tests testing differences in sampling position (tails vs. chest plumage). The effect of sampling method on disperser abundance (swab vs. direct feather sampling) was tested with the same method for chest plumage and tails separately. All statistical tests were performed in the R environment (R core team, 2020).

## 3 RESULTS

Diverse biological material was found attached to all three sampling positions in all sampled bird specimens using both the swab technique and direct extraction from feathers.

**FIGURE 2:**
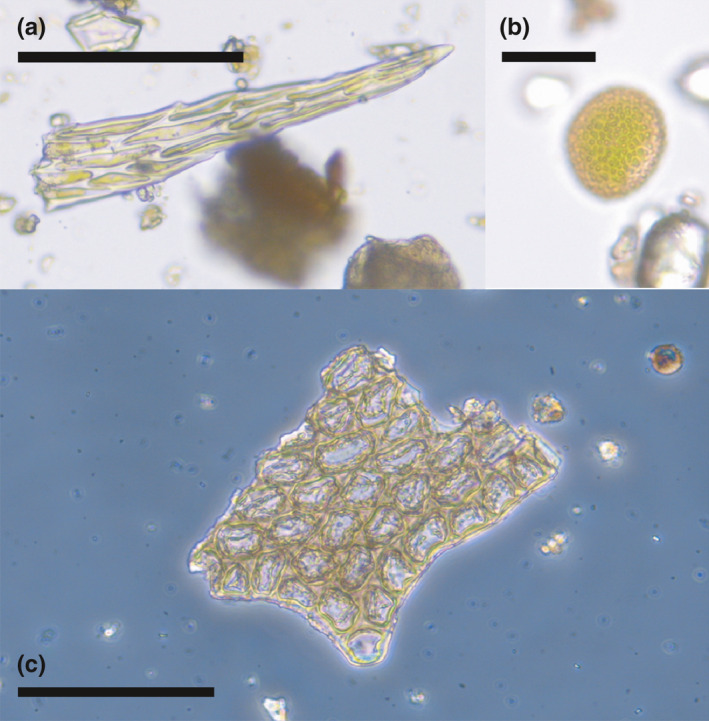
Plant diversity recovered from woodpecker specimens. (a) Leaf tip of moss. (b) Bryophyte spore. (c) Fragment of leaf margin of leafy liverwort, cf. Lophozia spp. Scale bars = 100 μm in A and C; 20 μm in B

Evidence of plants included leaf fragments of bryophytes, such as leaf tips of moss (Figure 2a), and fragments of liverwort leaves, including a fragment of leaf margin with well‐preserved tooth and laminal cells with trigones (Figure 2c). Bryophyte spores were also common in the samples (Figure 2b). Pollen grains were commonly recovered, including common forest trees in Finland (Pinus sylvestris, Picea abies, Betula spp., and Alnus spp.) and understory dwarf shrubs in the family Ericaceae.

**FIGURE 3:**
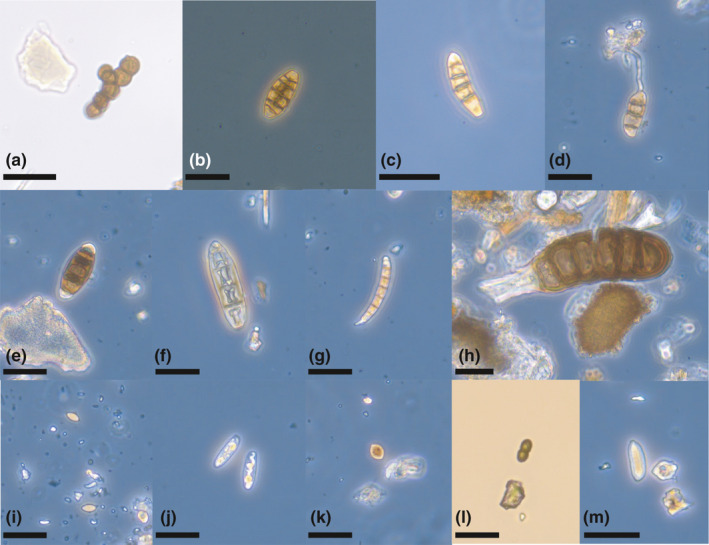
Fungal diversity recovered from woodpecker specimens. (a) Conidia of toruloid dematiaceous hyphomycetes. (b) Muriform conidium of dematiaceous hyphomycete. (c) Fungal conidium. (d) Germinating conidium. (e) Conidium of dematiaceous hyphomycete. (f) Conidium (aff. Drechslera or related genera). (g) Fusarium‐type conidium. (h) Multiseptate fragmenting conidium of dematiaceous hyphomycete. (i) Fusiform ascospores (potentially calicioid fungi). (j) Fungal spores (possibly basidiospores). (k) Fungal spore (possibly ascospore). (l) 1‐septate ascospore (potentially calicioid lichen). (m) Potential basidiospore with hilar appendage. All scale bars = 20 μm

Fungal diaspores were abundantly present, including both sexual spores and asexual conidia (Figure 3). The spores ranged from small to large (1─30 μm) and represented, for instance, globular, ellipsoidal, and fusiform nonseptate or one‐septate ascospores, as well as small unicellular and large, multiseptate, and muriform conidia. The latter are mostly produced by various dematiaceous hyphomycetes, tentatively including species from genera resembling Drechslera (Figure 3f) (Seifert et al., 2011). Fusarium‐type conidia were also recovered (Figure 3g). Some ascospores closely resemble those produced by calicioid lichens and fungi (Figure 3i,l). Potential basidiospores were also identified, with characteristic hilar appendage (Figure 3g).

**FIGURE 4:**
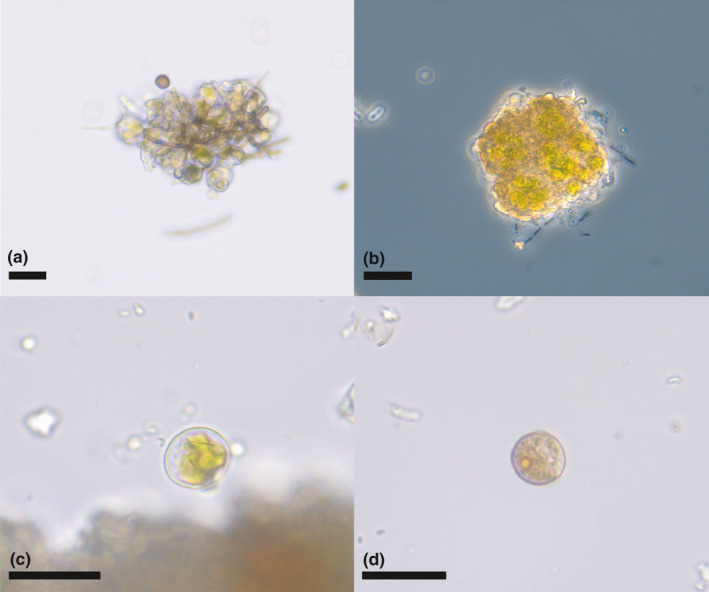
Algal diversity recovered from woodpecker specimens. (a‐b) Green algal cells within lichen soredia. (c) Green algal cell. (d) Green algal cell with pyrenoid. Scale bars = 20 μm

Lichen soredia (dispersal structures consisting of both fungal and photosynthetic green algal symbionts) were recovered from all bird specimens, sometimes in large quantities (Figure 4a,b). Also, free‐living green algae were recovered (Figure 4c,d).

**FIGURE 5:**
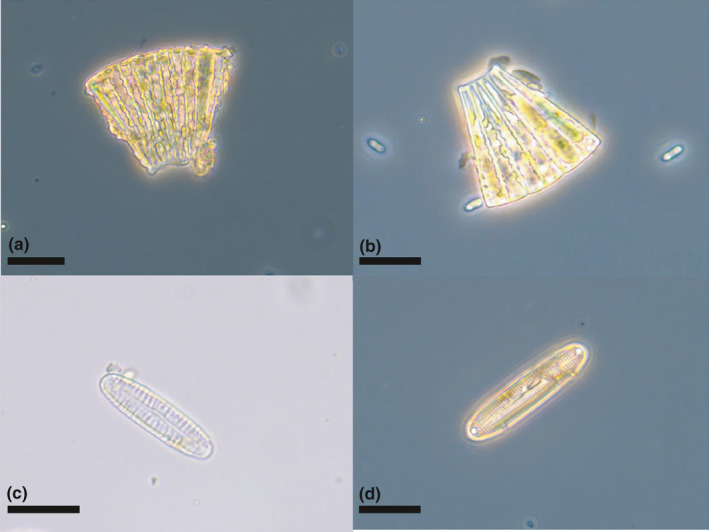
Diatom diversity recovered from woodpecker specimens. (a–b) Meridion circulare colonies. (c–d) Pinnularia spp. Scale bars = 20 μm

**FIGURE 6:**
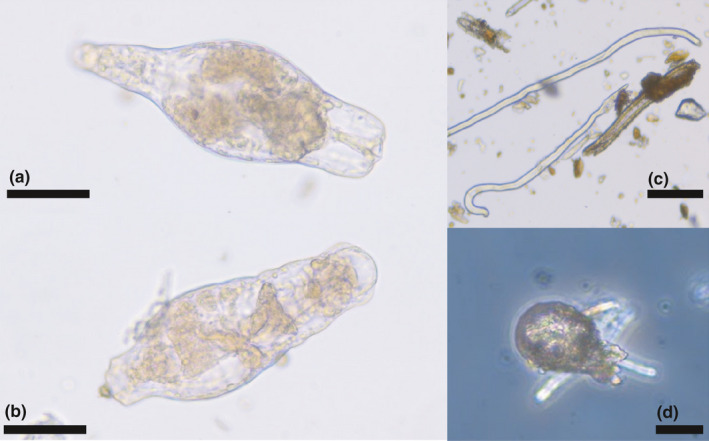
Microorganisms and animal diversity recovered from woodpecker specimens. (a–b) Rotifers. (c) Nematodes. (d) Testate ameba (possibly Difflugia sp.). Scale bars = 50 μm in a‐b, 100 μm in c, and 20 μm in d

Several diatoms were identified from the bird samples (Figure 5), including Meridion circulare colonies (Figure 5a,b) and several species of Pinnularia (Figure 5c,d). Other material identified included (partly decayed) rotifers (Figure 6a,b) nematodes (Figure 6c), testate amebae, possibly from the genus Difflugia (Figure 6d), and a broad range of other unidentified biological material, presumably mainly originating from vascular plants and arthropods (e.g., scales), as well as skin fragments shed by the birds. In addition, samples included various inorganic material, mainly sand and sediment particles.

**FIGURE 7:**
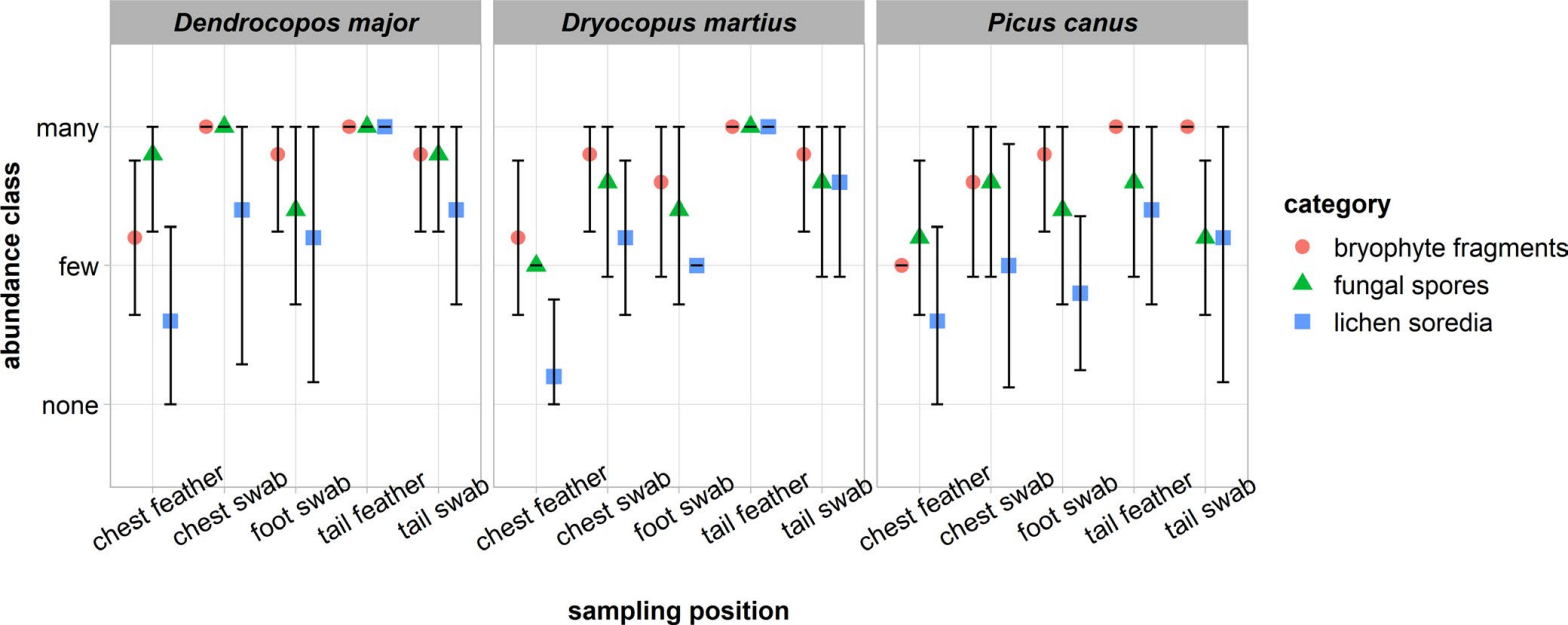
Mean abundance class estimates of three major categories of biological material found across the five sampling methods/positions in three species of woodpeckers (*N* = 15, five specimens per species, error bars = 95% confidence intervals). For the raw abundance estimates, see Appendix S2. The semiquantitative abundance estimates were coded as “none” (no material), “few” (1–5 units), and “many” (5+ units). The tails yielded a significantly higher total propagule load compared with chest plumage. Direct feather sampling yielded a significantly higher load in tail feathers compared with cotton swabs, the opposite is true for chest plumage

Bryophyte fragments, fungal spores, and lichen soredia were the most frequent material categories found. The abundance estimates of these categories are reported in Figure 7. The most abundant attached material was recovered from tail feathers directly, but all sampling positions and both sampling methods yielded ample biological material (Figure 7, Appendix S2). The combined abundance estimates using both methods from the tails were significantly higher than those from chest plumage (*W* = 5,618, *p* = 2.87e−07). In tails, the direct sampling method yielded a higher amount of material compared with cotton swabs (*W* = 740, *p* = .0016). In chest plumage, the opposite is true: Swabbing method recovered a higher yield (*W* = 510, *p* = 3.06e−06). There are no obvious differences across the three species of woodpeckers surveyed.

## 4 DISCUSSION

This study demonstrates that woodpecker tails, chest feathers, and feet, especially tails, can harbor a plethora of biological material originating from numerous different organisms from a wide taxonomical range. For example, we show, to our knowledge, the first evidence of freshwater diatoms and lichen soredia from the plumage of terrestrial birds. Our results confirm the findings of Chmielewski and Eppley (2019) who reported that bryophyte fragments are commonly found attached to birds. The material recovered is most probably a combination of material gathered from surfaces, material secondarily deposited from air, and resident species in feathers, for example, keratinophilic fungi (e.g., Labrador et al., 2020). The high amount of nonresident lichen soredia and bryophyte fragments highlights that material is picked up from surfaces, and this material is clearly most relevant for potential dispersal.

The diatom species most commonly encountered was Meridion circulare, which is thought to mainly occur attached to rocks in cold freshwater streams in Finland (Tikkanen, 1986). This makes it a surprising find in woodpecker feathers. It is possible that the diatom colonies attached to the bird during bathing (Simonis & Ellis, 2014), or the species might occur in other habitats where it has been previously undetected, such as ephemeral water bodies or bryophyte mats or soil in forest environments. These habitats would more likely allow the attachment to woodpecker feathers compared with freshwater rocks. Aerial dispersal of freshwater diatoms may be more common than previously thought (Bertrand et al., 2016), constituting yet another possible route of attachment. Similarly, the rotifers and testate amebae now observed from strictly terrestrial group of birds are known previously known only from water‐associated birds (Green & Elmberg, 2014). This study demonstrates that dispersal of freshwater diatoms and zooplankton by terrestrial birds is possible; however, likely it is not as important a dispersal avenue as freshwater‐associated birds, such as waterfowl (Coughlan et al., 2017; Fig uerola & Green, 2002; Green & Elmberg, 2014).

The common occurrence of lichen soredia in woodpecker plumage and the high load of fungal diaspores, possibly also from lichen‐forming fungi, highlight the potential of bird‐mediated dispersal of lichens. Woodpeckers are mostly resident in Finland, but occasionally show irruptive migration across Eurasia (Lindén et al., 2011). This allows potential dispersal not only on the local but also on the landscape or even continental scales. Woodpeckers could be especially important dispersal vectors for lichen species specializing on dead wood substrates, where woodpeckers forage and nest. Many dead wood‐associated lichens are endangered (Pykälä et al., 2019), and knowing about their dispersal interactions with other taxa is crucial for their effective conservation in fragmented forest landscapes.

Birds may facilitate the dispersal of the photosynthetic partner of the lichen symbiosis as well. This could be a key process to understand some of the special features of the dispersal ecology of symbiotic lichens. For instance, bird‐mediated photobiont dispersal may play a role in the relichenization process (the coming together of symbiotic partners). When a lichen mycobiont reproduces sexually, the dispersing fungal spore must encounter a suitable photobiont for the lichen to colonize a new habitat. Codispersal of symbiotic partners via, for example, soredia may circumvent this issue, but only very rarely lichens reproduce strictly with propagules allowing codispersal. Most lichen mycobionts are fertile at least occasionally, indicating that the relichenization process occurs in the life cycle of most lichens species known (Nash, 2008). Calicioid lichens (Caliciales) are an example of a diverse, cosmopolitan lichen group, which very rarely produce codispersing propagules (Tibell, 1999) and thus seem to rely solely on relichenization in their dispersal.

Long‐distance dispersal by migratory birds has been hypothesized to account for colonization of remote island systems (Dal Forno et al., 2017) and to explain disjunct biogeographical patterns present in many lichen species (Garrido‐Benavent & Perez‐Ortega, 2017). Phylogeographic analyses have demonstrated ongoing long‐distance gene flow in lichen mycobionts (Buschbom, 2007; Garrido‐Benavent et al., 2018; Myllys et al., 2003), but little direct evidence is available how this can be achieved. Airborne spores and vegetative dispersal units (including soredia) have been shown to be important in aerial dispersal (Harmata & Olech, 1991; Marshall, 1996; Muñoz et al., 2004). However, modeling studies indicate that it is exceedingly unlikely that airborne dispersal units would readily cross of the equator, especially for larger propagule sizes (Wilkinson et al., 2012). If further studies confirm the wide presence of lichen propagules in equator‐crossing migratory birds, long‐distance dispersal events by bird vectors, even if rare, could explain cases of observed trans‐equatorial gene flow (Geml et al., 2012).

The reproductive strategies and morphological traits of some lichen groups may facilitate spore attachment to feathers. For example, calicioid lichens produce stalked, mazaediate apothecia and are often referred to have a “spore‐saving” dispersal strategy (Tibell, 1994). Spores in Figure 3l are a close match to spores of many Calicium species, including taxa that have previously been suggested to be animal‐dispersed (Rikkinen, 1995). Based on amber fossils, the morphology of calicioid lichens and allied fungi has remained remarkably conserved since the Eocene (Rikkinen & Schmidt, 2018). Oldest woodpecker fossils from lineages with rigid tail feathers are from 22.5 Mya (de Pietri et al., 2011), suggesting that potential dispersal interactions may have been present already in the early Miocene.

One must emphasize that the presence of propagules on woodpecker feathers does not prove that effective woodpecker‐mediated dispersal occurs in nature but highlights the potential importance of such a process. The present evidence shows that many types of propagules are picked up by the woodpeckers and are also effectively transported to new locations, as some of our bird specimens were collected far from resident forests. There is evidence that bryophyte diaspores recovered from birds are mostly viable (Chmielewski & Eppley, 2019) and there is no reason to believe that propagules belonging to other taxa would not be. Propagules might even be better shielded from UV radiation when attached to bird feathers than when floating freely in the air. It remains for future studies to demonstrate successful establishment of lichenized fungi transported by birds to new sites. As aerial dispersal seems to result in successful establishment, we believe that bird‐mediated dispersal of lichens can often be effective—a result demonstrated in woodpecker‐mediated dispersal of nonlichenized fungi (Jusino et al., 2016).

The scale of bird‐mediated dispersal is notoriously difficult to quantify, especially for microscopic dispersers (Coughlan et al., 2019; Viana et al., 2016). Considering the high load of propagules in all the studied individuals and the commonness of woodpeckers in Finnish forests, the dispersal effect could be substantial. The relative importance of bird‐mediated dispersal compared with other dispersal avenues, such as wind dispersal, is likely taxon‐specific—more important for species with traits less suitable for other avenues of dispersal. Woodpecker‐mediated dispersal could be especially important for rare and highly substrate‐specific species, such as certain calicioid lichens (Rikkinen & Schmidt, 2018; Tibell, 1994) or aspen specialists (Gjerde et al., 2012).

The quantitative results here highlight that all of the birds sampled bear propagules in all of the sampling positions and that tails bear the highest amount of material. Both swabbing and direct feather sampling methods work, but the superior method is different in chest plumage and tails. This is not surprising, as swabbing the chest collects material from multiple feathers, whereas in tails, the material seems to be concentrated on retrix tips, easily sampled directly from museum specimens. For future studies, we recommend swabbing methods as they clearly work and are less invasive when working with live animals.

The method used here does not usually allow species‐specific identification of taxa since fungal spores or lichen soredia can rarely be confidently assigned to species with light microscopy. In future studies, molecular species identification based on metabarcoding methods (Abrego et al., 2018) would enable more accurate characterization of the full propagule assemblage attached to feathers and the identification of taxon‐specific dispersal interactions.

The potential of the described method extends beyond dispersal ecology to organismal biology wider. The chosen vector group can easily be expanded to other birds, mammals, and beyond, utilizing both existing natural history collections and live organisms. The traits, movement, or behavior of vector species and individuals, derived from specimens directly or indirectly (e.g., bird ringing details), could be linked to propagule loads (Chmielewski & Eppley, 2019; de Morais Junior et al., 2019). Additionally, knowledge about the dispersing organisms may be used to reveal new information about the movement and behavior of the vector species as well. Molecular methods, allowing not only propagule identification but also genotypification could yield interesting insights into movement ecology of many animals. If birds seem to effectively “sample” their surroundings, data from fresh or museum specimens may be used to reconstruct fungal, plant, animal, or microbial communities the birds encountered. These layers of information, extractable from natural history collections alone, emphasize their position as key sources of data. Collections have yet untapped potential for studies in not only systematics, but also ecology and organismal biology more widely.

## 5 CONCLUSIONS

Using natural history collections, we demonstrate for the first time a high amount of diverse microscopic propagules attached to woodpecker plumage. This highlights the potential of woodpeckers as dispersal vectors in fungi, including lichens, bryophytes, diatoms, algae, zooplankton, and various microorganisms. These potential bird dispersal interactions have diverse ecological consequences that may help explain patterns in species distribution, gene flow, and biogeography. The methods described here highlight the innovative use of natural history collections in ecology and organismal biology.

## CONFLICT OF INTEREST

None declared.

## AUTHOR CONTRIBUTIONS

**Niko R. Johansson:** Conceptualization (equal); investigation (equal); methodology (equal); writing–original draft (equal); writing–review and editing (equal). **Ulla Kaasalainen:** Conceptualization (equal); supervision (equal); writing–review and editing (equal). **Jouko Rikkinen:** Conceptualization (lead); supervision (equal); writing–review and editing (equal).

## Supporting information

Appendix S1

Appendix S2

## Data Availability

All specimen data can be accessed from Appendix S1. All raw abundance estimates can be accessed from Appendix S2. These datasets can also be accessed in Dryad: https://doi.org/10.5061/dryad.mgqnk98zs.
